# Relationships among physical activity, regulatory emotional self-efficacy, psychological detachment, and job burnout among urban workers

**DOI:** 10.3389/fpubh.2025.1589820

**Published:** 2025-06-18

**Authors:** Ke Xu, Hongyu Jiang, Huilin Wang

**Affiliations:** ^1^School of Physical Education, Hunan University of Science and Technology, Xiangtan, China; ^2^School of Business, Hunan University of Science and Technology, Xiangtan, China

**Keywords:** physical activity, regulatory emotional self-efficacy, psychological detachment, job burnout, urban young and middle-aged workers

## Abstract

**Introduction:**

Urban workers face a series of negative stimuli in the contemporary workplace, such as faster work pace, disproportionate income to effort, and job insecurity. These negative stimuli are often among the causes of job burnout in urban workers. Burnout among workers can negatively affect their families, businesses, and their own physical and mental well-being. Therefore, the emergence of burnout symptoms among urban workers has become a pressing issue. Addressing how to alleviate job burnout and improve their physical and mental well-being is the focus of this study.

**Methods:**

This cross-sectional study employed snowball and convenience sampling methods, collecting 395 valid responses from urban young and middle-aged workers across the public service, manufacturing, service, and information technology industries in Hunan, Jiangxi, and Anhui provinces, China. Structural equation modeling was performed using SmartPLS 4 to examine the proposed hypotheses.

**Results:**

The results indicated that physical activity was positively associated with regulatory emotional self-efficacy and psychological detachment (*β* = 0.446, *p* < 0.001; *β* = 0.343, *p* < 0.001, respectively). Furthermore, regulatory emotional self-efficacy was also positively related to psychological detachment (*β* = 0.494, *p* < 0.001). Both variables showed significant negative associations with job burnout (*β* = −0.307, *p* < 0.001; *β* = −0.400, *p* < 0.001, respectively). In addition, regulatory emotional self-efficacy and psychological detachment had a significant negative mediating effect on the relationship between physical activity and job burnout (standard indirect effect = −0.362, *p* < 0.001).

**Discussion:**

This implies that urban young and middle-aged workers who engage in physical activity are more likely to develop stronger regulatory emotional self-efficacy and psychological detachment, which in turn helps reduce job burnout. To better mitigate job burnout in urban young and middle-aged workers, governments, businesses, and individuals should emphasize the importance of physical activities.

## 1 Introduction

Job burnout arises from prolonged work-related stress, manifesting as emotional exhaustion, cynicism, and inefficacy (1, 2). Notably, the proportion of workers experiencing burnout rose to 60% following the outbreak of the COVID-19 pandemic (3). The COVID-19 pandemic also triggered widespread socio-economic disruptions (4), exacerbating job insecurity, income inequality, and health issues among urban young and middle-aged workers—a group particularly vulnerable to rapid work changes and ongoing uncertainties (5, 6). These evolving work demands negatively impact workers’ resilience and effectiveness, thereby contributing to burnout (6, 7).

Given the widespread nature of burnout, it is crucial to understand not only its causes but also its broader consequences on individuals and organizations. Burnout has been shown to undermine workers’ emotional, psychological, and behavioral functioning. Some resort to harmful coping mechanisms—such as increased smoking or alcohol consumption—to alleviate burnout-related stress (1). Additionally, job burnout is associated with rising absenteeism, turnover rates, and decreased job performance (8). The negative consequences extend beyond individuals, impacting their families, work environments, and broader organizational arrangements (2, 9). Severe burnout also impairs physical and mental well-being, with symptoms such as reduced self-efficacy, poor sleep, cognitive decline, and a higher risk of cardiovascular diseases (10).

Amid these challenges, physical activity has emerged as a protective factor. Studies show that regular physical activity boosts self-efficacy, reduces fatigue, and alleviates workplace stress (10–12). Employees who consistently engage in physical activities tend to exhibit greater motivation and perceive job demands as less overwhelming (13–15). Physical activity—broadly defined as any skeletal muscle-induced movement that results in energy expenditure—encompasses both general movement and structured exercise (16), with well-established physical and mental health benefits such as reducing obesity, type 2 diabetes, and cardiovascular risk (17–19). Conversely, physical inactivity has been linked to higher rates of burnout and depression, reinforcing a vicious cycle of stress and withdrawal (20). Participation in physical activity enhances personal competence, fosters emotional self-regulation, and reduces sensitivity to negative stimuli, thus mitigating both burnout and depression (20–22).

Although previous research has explored the psychological benefits of physical activity, it has primarily focused on individuals pursuing exercise to achieve task goals or improve performance (23). However, urban young and middle-aged workers often engage in physical activity for stress relief and relaxation rather than performance enhancement. In addition, prior studies examining psychological detachment often assume that workers naturally detach during non-work hours (24). In contrast, this study emphasizes the proactive role of physical activity in facilitating psychological detachment during leisure time. Therefore, this study aims to (1) assess the relationships among physical activity, regulatory emotional self-efficacy, psychological detachment, and burnout in urban young and middle-aged workers; (2) explore the mediating roles of regulatory emotional self-efficacy and psychological detachment; and (3) propose strategies to enhance well-being and work efficiency in this population.

Beyond emotional resources, physical activity also facilitates recovery from work stress through psychological detachment. According to Attention Restoration Theory, restorative environments promote recovery from attentional fatigue (25), and physical activity often takes place in such environments (26). Psychological detachment refers to the mental disconnection from work during non-working hours, allowing individuals to “switch off” work-related thoughts (27). When individuals immerse themselves in non-work activities—such as leisure-time physical activity—they are more likely to achieve psychological detachment, which in turn reduces work-related stress and contributes to better mental health outcomes (24, 28).

Furthermore, regulatory emotional self-efficacy and psychological detachment have been identified as mediators between job stressors and burnout. Individuals who believe in their ability to regulate emotions can neutralize negative affect and maintain job satisfaction (29, 30). Likewise, detachment allows for mental distance from job demands, particularly when engaging in restorative activities such as physical exercise (31–33). Studies also suggest that physical activity positively affects both emotional self-efficacy and detachment (13, 34), and through these mechanisms, indirectly reduces job burnout. Long-term participation in physical activities has been shown to enhance body image and personal confidence (35), while enjoyable leisure experiences provide opportunities to mentally disengage from work (36–38). Based on the above literature, this study proposes the following hypotheses:

*H1:* Physical activity is positively associated with regulatory emotional self-efficacy.

*H2:* Physical activity is positively associated with psychological detachment.

*H3:* Regulatory emotional self-efficacy is positively associated with psychological detachment.

*H4:* Regulatory emotional self-efficacy is negatively associated with job burnout.

*H5:* Psychological detachment is negatively associated with job burnout.

*H6:* Regulatory emotional self-efficacy and psychological detachment mediate the relationship between physical activity and job burnout.

**Figure 1:**
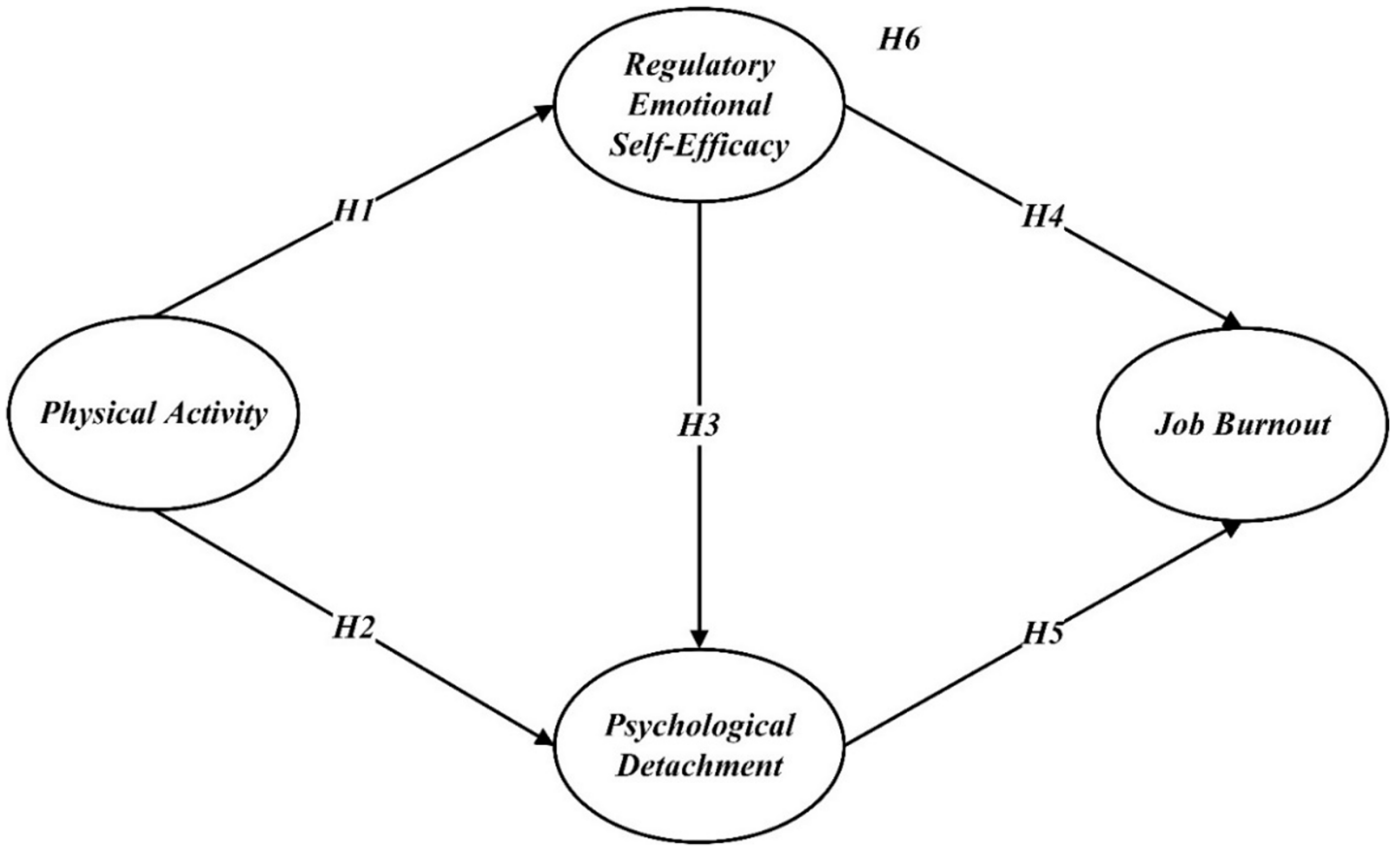
Hypothetical model.

A summary of all hypotheses is illustrated in Figure 1.

## 2 Methodology

### 2.1 Participants and procedures

This study employed convenience sampling and snowball sampling methods to conduct a questionnaire survey among urban young and middle-aged workers (aged 18–44) across various industries. Following the age group definitions proposed by Mullet et al. (39) and Monod et al. (40), individuals aged 18–34 were classified as young adults, and those aged 35–44 as middle-aged adults, forming the target population of this study.

To collect relevant research data, a questionnaire survey was conducted between December 2024 and January 2025 among urban workers in Hunan, Jiangxi, and Anhui provinces, China. The survey covered industries such as public services, manufacturing, service industries, and information technology. The survey was distributed through social platforms like Tencent QQ and WeChat, inviting potential participants to join. Those who agreed received a survey link and were informed about the study’s purpose and voluntary participation. As appreciation, participants received a sports shower gel or a sports app voucher and were encouraged to share the link with colleagues and friends.

A total of 433 questionnaires were distributed, with 395 valid responses retained after excluding those with rapid completion times or identical answer patterns, yielding a 91.2% valid response rate. Table 1 summarizes the demographic characteristics of the respondents: (1) Gender distribution was nearly equal, with 50.9% male and 49.1% female participants. (2) Around 45% of respondents were aged 18–24. (3) Around 43.8% of participants worked in the public service sector, including education, healthcare, and government. (4) Nearly 46.3% of respondents reported a monthly income between 5,001 and 10,000 CNY.

**Table 1:** Demographic characteristics.

Category		*n*	%
Gender	Male	201	50.9
	Female	194	49.1
Age group	18–24	178	45.1
	25–34	149	37.7
	35–44	68	17.2
Highest education level	High school or below	61	15.4
	Associate degree	112	28.4
	Bachelor’s degree	179	45.3
	Master’s degree or above	43	10.9
Industry	Public services (e.g., education, healthcare, government)	173	43.8
	Manufacturing	43	10.9
	Service industry (e.g., retail, hospitality, catering)	31	7.8
	Information technology	62	15.7
	Other	86	21.8
Monthly income (CNY)	≤5,000	171	43.3
	5,001–10,000	183	46.3
	10,001–15,000	21	5.3
	≥15,001	20	5.1
Self-reported health status	Good	284	71.9
	Average	105	26.6
	Poor	6	1.5

### 2.2 Instruments

The questionnaire consisted of five sections. The first collected demographic data, including age, gender, industry, income, and self-reported health. The second section assessed physical activity levels using a three-item scale adapted by Chinese scholar Liang Deqing, as previously employed in the study Ren et al. (41). Responses were measured on a five-point Likert scale. This scale, originally developed in Chinese, aligns well with China’s social and cultural context and has been widely adopted in numerous studies, including those by Wang et al. (42), Li et al. (43), and Zou et al. (44). An example item was: “What is the intensity of physical activity that you usually participate in during the past month?” Each item was rated on a five-point scale to facilitate quantitative analysis. For instance, responses to the physical activity intensity question included: light physical activity (1 point), light-to-moderate physical activity (2 points), moderate-to-high intensity physical activity (3 points), high-intensity but short-duration physical activity (4 points), and high-intensity and long-duration physical activity (5 points). The third assessed regulatory emotional self-efficacy with eight items from Caprara et al. (45), such as: “Manage negative feelings when reprimanded by your parents or significant others.” The fourth measured psychological detachment using four items from Shimazu et al. (46), including: “I get a break from the demands of work.” The fifth evaluated job burnout with seven items from Kristensen et al. (47), such as: “Are you exhausted in the morning at the thought of another day at work?” Except for the physical activity measurement, all responses were on a five-point Likert scale (1 = strongly disagree to 5 = strongly agree).

### 2.3 Data analysis

This study employed structural equation modeling (SEM) using SmartPLS 4 to analyze the proposed model. SEM is widely used to assess latent variables and test their relationships (48). Following Anderson and Gerbing (49) two-step approach, both measurement and structural models were evaluated. Reliability and validity were first assessed, followed by an analysis of the relationships among physical activity, regulatory emotional self-efficacy, psychological detachment, and job burnout using maximum likelihood estimation. Bootstrapping with 5,000 samples tested indirect effects, and model fit indices and path coefficients were examined.

## 3 Results

### 3.1 Assessment of the measurement model reliability and validity

In the initial phase of the PLS-SEM analysis, Cronbach’s alpha (Cα), composite reliability (CR), and rho-A were used to assess internal reliability, with all constructs demonstrating satisfactory consistency. The lowest Cα was 0.898, and the CR was 0.937. Convergent validity was assessed using average variance extracted (AVE), requiring a minimum threshold of 0.50. All AVE scores met this criterion, confirming sufficient convergent validity. Table 2 presents the results for Cα, CR, and AVE.

**Table 2:** Reliability and validity test.

Items	Loading	Cα	CR	AVE
**Physical activity (PA)**		0.898	0.937	0.831
PA1	0.923			
PA2	0.915			
PA3	0.896			
**Regulatory emotional self-efficacy (RES)**		0.965	0.970	0.803
*Perceived self-efficacy in managing despondency/distress (DES)*		0.950	0.964	0.869
DES1	0.922			
DES2	0.925			
DES3	0.943			
DES4	0.939			
*Perceived self-efficacy in managing anger/irritation (ANG)*		0.947	0.962	0.864
ANG1	0.927			
ANG2	0.924			
ANG3	0.944			
ANG4	0.921			
**Psychological detachment (PD)**		0.929	0.950	0.826
PD1	0.921			
PD2	0.922			
PD3	0.935			
PD4	0.855			
**Job burnout (JB)**		0.961	0.968	0.811
JB1	0.866			
JB2	0.914			
JB3	0.903			
JB4	0.875			
JB5	0.907			
JB6	0.903			
JB7	0.935			

Cα, Cronbach’s alpha; AVE, average variance extracted; CR, composite reliability.

Additionally, discriminant validity for all factors was established through discriminant testing. This form of validity requires that components be statistically unrelated to other factors when assessing measurement correspondences. Discriminant validity was assessed by comparing the square root of the AVE with factor correlations, requiring the square root of the AVE to be greater than the correlation values. The results showed that all AVE square roots exceeded the correlation coefficients (Table 3), confirming strong discriminant validity. Additionally, the variance inflation factor (VIF) was used to check multicollinearity, with all VIF values below the acceptable threshold of 3.3. In this study, all VIF values were under 1.6, indicating that multicollinearity is not a concern.

**Table 3:** Display discriminant validity analysis.

Items	RES	JB	PA	PD
RES	0.896			
JB	−0.565	0.901		
PA	0.446	−0.543	0.912	
PD	0.647	−0.598	0.563	0.909

PA, physical activity; RES, regulatory emotional self-efficacy; PD, psychological detachment; JB, job burnout.

### 3.2 Hypothesis testing results

The model fit was assessed using the standardized root mean square residual (SRMR), a standardized residuals index that compares the observed covariance with the hypothesized matrix. SRMR values of 0.08 or lower are generally deemed acceptable. The estimated SRMR value in this study was 0.046, indicating a satisfactory model fit.

**Figure 2:**
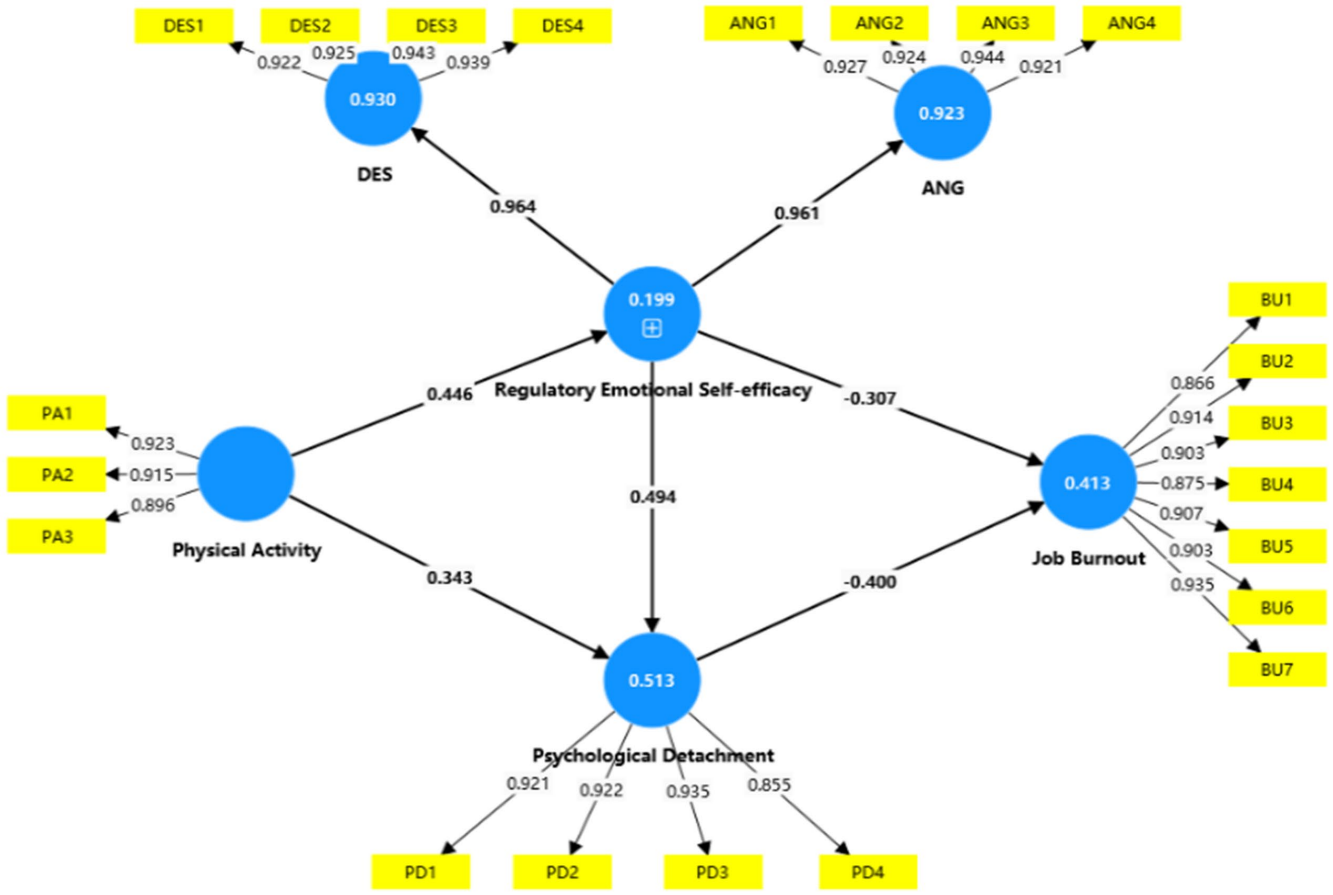
Structural path model. PA, physical activity; DES, perceived self-efficacy in managing despondency/distress; ANG, perceived self-efficacy in managing anger/irritation; PD, psychological detachment; JB, job burnout.

As shown in Table 4 and Figure 2, the structural path model revealed that physical activity was significantly positively associated with regulatory emotional self-efficacy (*β* = 0.446, *t* = 11.479, *p* < 0.001) and psychological detachment (*β* = 0.343, *t* = 7.614, *p* < 0.001), supporting H1 and H2. Regulatory emotional self-efficacy was significantly positively associated with psychological detachment (*β* = 0.494, *t* = 9.791, *p* < 0.001) and significantly negatively associated with job burnout (*β* = −0.307, *t* = 4.726, *p* < 0.001), supporting H3 and H4. In addition, psychological detachment was also significantly negatively associated with job burnout (*β* = −0.400, *t* = 6.112, *p* < 0.001), supporting H5.

**Table 4:** Path coefficients.

No.	Path	*β*	SEs	*T* statistics	*p* values	LLIC	ULIC
H1	PA - > RES	0.446	0.447	11.479	0.000	0.368	0.522
H2	PA - > PD	0.343	0.345	7.614	0.000	0.258	0.432
H3	RES - > PD	0.494	0.493	9.791	0.000	0.392	0.591
H4	RES - > JB	−0.307	−0.307	4.726	0.000	−0.438	−0.186
H5	PD - > JB	−0.400	−0.401	6.112	0.000	−0.523	−0.272

PA, physical activity; RES, regulatory emotional self-efficacy; PD, psychological detachment; JB, job burnout.

### 3.3 Mediation analysis

The study hypothesized that physical activity affects job burnout through two mediators: regulatory emotional self-efficacy and psychological detachment. Mediation effects were tested using bootstrapping (50). Table 5 presents standardized results from 5,000 bootstrap samples with 95% confidence intervals, showing no zero values. Regulatory emotional self-efficacy and psychological detachment significantly mediated the relationship between physical activity and job burnout (standard indirect effect = −0.362, *p* < 0.001), supporting H6. Findings suggest that higher physical activity levels enhance these mediators, leading to lower job burnout.

**Table 5:** Mediation analysis.

No	Path	Effect	Boot SE	*T* statistics	*P* values	Boot LLIC	Boot ULIC
H6	Total indirect effect of PA - > JB	−0.362	−0.365	9.185	0.000	−0.44	−0.287
	Total effect of PA - > JB	−0.362	−0.365	9.185	0.000	−0.44	−0.287

PA, physical activity; JB, job burnout.

## 4 Discussion

### 4.1 Theoretical contributions

This study aimed to examine the relationships among physical activity, regulatory emotional self-efficacy, psychological detachment, and job burnout among young and middle-aged workers, with a particular focus on the mediating roles of regulatory emotional self-efficacy and psychological detachment. The findings revealed that both regulatory emotional self-efficacy and psychological detachment were negatively associated with job burnout. This supports the arguments of Alessandri et al. (51) and Muhamad Nasharudin et al. (52), who suggested that the greater an individual’s ability to regulate negative emotions and the higher their level of psychological detachment, the more resilient they are under pressure. Furthermore, psychological detachment may enhance well-being, thereby reducing the risk of burnout. The results also showed a significant positive relationship between regulatory emotional self-efficacy and psychological detachment, aligning with the findings of Sheppes et al. (53), who argued that individuals with stronger emotion regulation abilities are more likely to adopt effective emotional coping strategies. For example, when facing intense negative situations, such individuals tend to disengage and use distraction techniques to manage their emotions.

In addition, this study applied Attention Restoration Theory to explore how physical activity influences job burnout among urban young and middle-aged workers. However, previous studies applying Attention Restoration Theory to workplace settings have primarily focused on the role of natural environments in alleviating work-related fatigue, exploring their restorative benefits in detail. For instance, solo camping in the wilderness has been found to help workers shift their attention away from high-pressure situations, thereby promoting mental clarity and reducing fatigue (54, 55). However, few scholars have investigated the restorative effects of exercise environments. Building on this foundation, the present study integrates prior findings on fatigue—as a key antecedent of job burnout (56) —to examine how physical activity environments contribute to alleviating burnout among urban young and middle-aged workers, thus expanding the application boundaries of Attention Restoration Theory.

In addition, previous research has predominantly focused on mitigating job burnout by changing workers’ job behaviors or enhancing internal psychological resources (57, 58). Yet, limited attention has been paid to the role of physical activity in reducing burnout in this population, or to its potential mediating effects through regulatory emotional self-efficacy and psychological detachment. Therefore, this study highlights the mediating roles of regulatory emotional self-efficacy and psychological detachment. The findings suggest that physical activity can enhance these mediators, thereby reducing job burnout among urban young and middle-aged workers.

In earlier studies, Valois et al. (23) suggested that placing individuals in high-goal contexts (e.g., competitive sports settings) may induce anxiety and reduce regulatory emotional self-efficacy. This conclusion stemmed from research in which individuals engaged in physical activity primarily to accomplish specific tasks or enhance athletic performance. In contrast, the urban young and middle-aged workers in this study participate in physical activity during their non-working hours with the aim of relaxation and fatigue relief. As such, the results demonstrate a positive association between physical activity and regulatory emotional self-efficacy, likely because physical activity reduces negative emotions and enhances one’s sense of personal efficacy (35).

Furthermore, earlier studies have found that high-pressure work environments and ongoing work demands often lead to stress spillover into non-working hours, making psychological detachment difficult (59, 60). These findings were based on contexts in which the boundary between work and non-work time was blurred. In contrast, participants in this study engage in physical activity within freely chosen leisure environments during their off-duty hours, which facilitates greater psychological detachment.

Previous research has also reported that psychological detachment positively influences physical activity, typically assuming that workers immediately disengage from work upon finishing their shifts (24). However, in the current study, workers actively choose to participate in physical activity after work, which also enhances their level of psychological detachment. This supports our hypothesis that physical activity positively contributes to psychological detachment, potentially due to its role in alleviating attentional fatigue (26).

### 4.2 Practical implications

This study examined the associations among physical activity, regulatory emotional self-efficacy, psychological detachment, and job burnout among urban young and middle-aged workers. The findings indicated that physical activity was positively related to both regulatory emotional self-efficacy and psychological detachment, suggesting that it may help urban workers manage negative emotions and work-related stress, thereby potentially alleviating job burnout. Given that well-designed urban spaces and built environments contribute to healthier social structures and effectively promote and sustain physical activity (61, 62), this study proposes a three-tiered set of recommendations to encourage physical activity among urban workers: at the government level, the organizational level, and the individual worker level.

At the government level, policymakers should prioritize the development of accessible sports and recreational facilities in urban areas, particularly in proximity to workplace clusters. As work demands intensify, urban workers often experience physical and mental fatigue, leaving them with limited time and energy for physical activity. The establishment of sports venues or exercise spaces near workplaces can provide convenient opportunities for employees to engage in physical activities, thus facilitating participation and fostering a more active workforce. Additionally, increasing the availability of public sports equipment and promoting urban green spaces for exercise can further encourage physical activity in daily routines. Creating more comfortable and suitable outdoor environments, such as expanding urban green spaces, ensuring well-lit walking and jogging paths, and integrating physical activity-friendly designs into public spaces, is essential. Despite the potential of urban areas to promote physical activity, many spaces remain underutilized. Urban planning should incorporate exercise-friendly environments to maximize opportunities for movement and active living.

At the enterprise level, fostering a workplace culture that supports physical activity can contribute to employees’ well-being. According to ecosystem theory, the surrounding environment significantly shapes individual behavior and responses (63). Extending this view, Laustsen (63) emphasizes that the relationship between individuals and their environments is not unidirectional, but rather dynamic and reciprocal. In this sense, behavior is seen as an ecological phenomenon—emerging through continuous interactions between the person and the broader system. Applying this perspective to the workplace context highlights the importance of creating supportive environments that encourage physical activity. A workplace culture that integrates physical activity can enhance psychological resilience and stress management. Employers can cultivate such a culture by organizing workplace fitness programs, team-based physical activities, and incentive-driven sports competitions. In addition, as well-designed built environments have been shown to positively influence physical activity and overall health (64), companies may consider providing dedicated fitness spaces within office buildings to help employees better integrate physical activity into their daily routines.

At the individual level, since the built environment is a key factor influencing physical activity (65), workers can choose to live in areas with good exercise facilities to help cultivate sustainable exercise habits. Physical activity not only contributes to physical and mental well-being but also fosters a sense of social belonging and community integration. Engaging in group activities, such as running clubs or fitness classes, may further enhance motivation and social connections, reinforcing the benefits of an active lifestyle. To balance work and life effectively, individuals can set personal fitness goals, integrate active commuting (such as cycling or walking) into their daily routine, and schedule regular exercise sessions to ensure consistency. Time management skills, such as prioritizing workouts during lunch breaks or early mornings, can help sustain physical activity despite a busy work schedule. Mindfulness practices, such as combining exercise with relaxation techniques like yoga or meditation, can further support overall well-being and stress relief.

In conclusion, promoting physical activity among urban young and middle-aged workers has implications beyond individual well-being, extending to workplace productivity, societal health, and urban sustainability. Governments, enterprises, and individuals should collectively recognize the value of physical activity and implement supportive measures to integrate it into daily urban life. By fostering a physically active workforce and optimizing urban environments to encourage movement, we can contribute to healthier, more resilient urban communities.

### 4.3 Limitations

First, this study employed snowball sampling and convenience sampling to collect data from three provinces in central China. While these methods reduced the complexity of data collection, they may have limited the representativeness of the findings. Future research is encouraged to adopt random sampling methods to expand the geographical and occupational scope of data collection, thereby enhancing the generalizability and representativeness of the results.

Second, this study adopted a cross-sectional design, which lacks a temporal dimension and does not allow for causal inference. Future studies could consider longitudinal or intervention-based research designs. These approaches would provide deeper insights into the dynamic and causal relationships between physical activity and job burnout among urban young and middle-aged workers, allowing researchers to better capture trends and changes over time.

Third, physical activity levels were measured using a three-item scale adapted by Chinese scholar Liang Deqing, as employed in the study by Ren et al. (41). This scale was selected for its alignment with Chinese cultural and contextual characteristics. While this scale is contextually appropriate, self-reported measures may still be subject to recall bias, social desirability bias, or under−/over-reporting of physical activity levels. Future studies could incorporate wearable activity trackers (e.g., accelerometers) to obtain more objective and accurate data on physical activity intensity, thus improving the reliability of the measurements and offering a clearer understanding of urban workers’ activity levels.

Finally, this study focused on examining the mediating roles of regulatory emotional self-efficacy and psychological detachment in the relationship between physical activity and job burnout among urban young and middle-aged workers. Although this offers valuable insights, the study did not account for potential covariates—such as sleep quality, chronic health conditions, or working hours—which may influence this relationship. Future research could incorporate these covariates as moderators to further explore their impact and provide a more comprehensive understanding of the factors affecting job burnout.

## 5 Conclusion

The findings of this study align with its research objectives, highlighting the significant role of physical activity, regulatory emotional self-efficacy, and psychological detachment in alleviating job burnout among urban young and middle-aged workers. Furthermore, the results reveal that physical activity mitigates job burnout through the mediating effects of regulatory emotional self-efficacy and psychological detachment.

Therefore, this study advocates for urban young and middle-aged workers to actively engage in physical activities outside of work, as this enhances their regulatory emotional self-efficacy and psychological detachment levels. These factors play a crucial role in helping workers regulate their emotional state in response to negative stimuli, ultimately reducing the occurrence of job burnout symptoms.

Additionally, this study calls for government, businesses, and individual workers to take proactive measures in promoting physical activity. Governments should increase investment in the construction of sports facilities and venues in areas with high concentrations of enterprises. Businesses should foster a sports-friendly workplace culture and actively encourage employees to participate in physical activities. Urban young and middle-aged workers should develop the habit of engaging in regular physical activity, as it is essential for maintaining their physical and mental well-being and sustaining an optimal work state.

## Data Availability

The raw data supporting the conclusions of this article will be made available by the authors, without undue reservation.
