# Supplementary material for: Relationships among physical activity, regulatory emotional self-efficacy, psychological detachment, and job burnout among urban workers
Source: Front Public Health. 2025 Jun 18;13:1589820. doi: 10.3389/fpubh.2025.1589820 (PMC12213814; doi:10.3389/fpubh.2025.1589820)
Supplement: Supplementary file 1 [file Data_Sheet_1.pdf]

Questionnaire on the Relationship of Physical Activity to Emotion Regulation Self-Efficacy, Psychological Disengagement, and Burnout in Urban Young Adult Workers

Hello, dear gentlemen and ladies! First of all, thank you for your willingness to participate in our questionnaire! The purpose of this questionnaire is to understand the relationship between physical activity and your emotional regulation self-efficacy, psychological disconnection and burnout. All questions are not right or wrong, so please answer them as you see fit. The data collected will be used for academic research only, and we will keep your information strictly confidential, so please feel free to answer. Thank you again for your support of our research!

Variant	Subject	Mark
Demographic Information	What is your gender?	1. Male<br>2. Female
	What is your age?	1.18-24 years old<br>2. 25-34 years old<br>3. 35-44 years old
	What is your highest level of education?	1. High school or below<br>2. Associate degree<br>3. Bachelor's degree<br>4. Master's degree or above
	What is your current work industry?	1. Public services (e.g. education, healthcare, government)<br>2. Manufacturing<br>3. Service industry (e.g., retail, hospitality, catering)<br>4. Information Technology<br>5. Other

	What is your current monthly income level?	1. Less than 5000 yuan<br>2. 5001-10000 yuan<br>3. 10001-15000 yuan<br>4. More than 15,001 yuan
	How would you rate your physical health?	1. Good<br>2. Average<br>3. Poor
Physical Activity	What is the intensity of physical activity (PA) that you usually participate in during the past month?	1.Light-intensity physical activity (go for a walk and radio broadcast exercises, etc.)<br>2.Light-to moderate-intensity physical activities (recreational volleyball, table tennis, jogging and Tai Chi, etc.)<br>3.Moderate-to vigorous-intensity physical activities (cycling, running, and table tennis, etc.)<br>4.Vigorous but not lasting physical activities with sweating a lot (badminton. volleyball, basketball, tennis, and football, etc.)<br>5.Vigorous and lasting physical activities with sweating a lot (racing, aerobics practicing, and swimming, etc.)
	How long do you spend in each physical activity session?	1. Less than 10 min<br>2. 11-20 min<br>3. 21-30 min<br>4. 31-59 min<br>5. More than 60 min
	How often do you do physical activity every month/week?	1. Less than 1 time/month<br>2. 2-3 times/month<br>3. 1-2 times/week<br>4. 3-5 times/week

		5. Every day
Regulatory Emotional Self-Efficacy	Keep from getting dejected when you are lonely.	1. Strongly disagree<br>2. Quite disagree<br>3. Fairly agree<br>4. Quite agree<br>5. Strongly agree
	Keep from getting discouraged by strong criticism.	1. Strongly disagree<br>2. Quite disagree<br>3. Fairly agree<br>4. Quite agree<br>5. Strongly agree
	Reduce your upset when you don't get the appreciation you feel you deserve.	1. Strongly disagree<br>2. Quite disagree<br>3. Fairly agree<br>4. Quite agree<br>5. Strongly agree
	Keep from getting discouraged in the face of difficulties.	1. Strongly disagree<br>2. Quite disagree<br>3. Fairly agree<br>4. Quite agree<br>5. Strongly agree
	Manage negative feelings when reprimanded by your parents or significant others.	1. Strongly disagree<br>2. Quite disagree<br>3. Fairly agree<br>4. Quite agree<br>5. Strongly agree

	Avoid getting upset when others keep giving you a hard time.	1. Strongly disagree<br>2. Quite disagree<br>3. Fairly agree<br>4. Quite agree<br>5. Strongly agree
	Get over irritation quickly for wrongs you have experienced.	1. Strongly disagree<br>2. Quite disagree<br>3. Fairly agree<br>4. Quite agree<br>5. Strongly agree
	Avoid flying off the handle when you get angry.	1. Strongly disagree<br>2. Quite disagree<br>3. Fairly agree<br>4. Quite agree<br>5. Strongly agree
Psychological Detachment	I forget about work.	1. Strongly disagree<br>2. Quite disagree<br>3. Fairly agree<br>4. Quite agree<br>5. Strongly agree
	I don’t think about work at all.	1. Strongly disagree<br>2. Quite disagree<br>3. Fairly agree<br>4. Quite agree<br>5. Strongly agree

	I distance myself from my work.	1. Strongly disagree<br>2. Quite disagree<br>3. Fairly agree<br>4. Quite agree<br>5. Strongly agree
	I get a break from the demands of work.	1. Strongly disagree<br>2. Quite disagree<br>3. Fairly agree<br>4. Quite agree<br>5. Strongly agree
Job Burnout	Do you feel worn out at the end of the working day?	1. Strongly disagree<br>2. Quite disagree<br>3. Fairly agree<br>4. Quite agree<br>5. Strongly agree
	Are you exhausted in the morning at the thought of another day at work?	1. Strongly disagree<br>2. Quite disagree<br>3. Fairly agree<br>4. Quite agree<br>5. Strongly agree
	Do you feel that every working hour is tiring for you?	1. Strongly disagree<br>2. Quite disagree<br>3. Fairly agree<br>4. Quite agree<br>5. Strongly agree

	Do you feel that you don't have enough energy for family and friends during leisure time?	1. Strongly disagree<br>2. Quite disagree<br>3. Fairly agree<br>4. Quite agree<br>5. Strongly agree
	Is your work emotionally exhausting?	1. Strongly disagree<br>2. Quite disagree<br>3. Fairly agree<br>4. Quite agree<br>5. Strongly agree
	Does your work frustrate you?	1. Strongly disagree<br>2. Quite disagree<br>3. Fairly agree<br>4. Quite agree<br>5. Strongly agree
	Do you feel burnt out because of your work?	1. Strongly disagree<br>2. Quite disagree<br>3. Fairly agree<br>4. Quite agree<br>5. Strongly agree
